# Predicting Onset of Dementia Using Clinical Notes and Machine Learning: Case-Control Study

**DOI:** 10.2196/17819

**Published:** 2020-06-03

**Authors:** Christopher A Hane, Vijay S Nori, William H Crown, Darshak M Sanghavi, Paul Bleicher

**Affiliations:** 1 OptumLabs Optum Cambridge, MA United States

**Keywords:** Alzheimer disease, dementia, health information systems, machine learning, natural language processing, health information interoperability

## Abstract

**Background:**

Clinical trials need efficient tools to assist in recruiting patients at risk of Alzheimer disease and related dementias (ADRD). Early detection can also assist patients with financial planning for long-term care. Clinical notes are an important, underutilized source of information in machine learning models because of the cost of collection and complexity of analysis.

**Objective:**

This study aimed to investigate the use of deidentified clinical notes from multiple hospital systems collected over 10 years to augment retrospective machine learning models of the risk of developing ADRD.

**Methods:**

We used 2 years of data to predict the future outcome of ADRD onset. Clinical notes are provided in a deidentified format with specific terms and sentiments. Terms in clinical notes are embedded into a 100-dimensional vector space to identify clusters of related terms and abbreviations that differ across hospital systems and individual clinicians.

**Results:**

When using clinical notes, the area under the curve (AUC) improved from 0.85 to 0.94, and positive predictive value (PPV) increased from 45.07% (25,245/56,018) to 68.32% (14,153/20,717) in the model at disease onset. Models with clinical notes improved in both AUC and PPV in years 3-6 when notes’ volume was largest; results are mixed in years 7 and 8 with the smallest cohorts.

**Conclusions:**

Although clinical notes helped in the short term, the presence of ADRD symptomatic terms years earlier than onset adds evidence to other studies that clinicians undercode diagnoses of ADRD. De-identified clinical notes increase the accuracy of risk models. Clinical notes collected across multiple hospital systems via natural language processing can be merged using postprocessing techniques to aid model accuracy.

## Introduction

### Background

Worldwide, up to 77% of people with dementia are undiagnosed, and “lack of detection is a significant barrier to improving the lives of people with Alzheimer’s disease and other dementias, their families and careers” [1]. This also implies that more than three-quarters of the patient population with dementia is not being referred for participation in clinical trials to study new potential treatments for neurodegenerative diseases. There are many factors influencing clinical trial recruitment for Alzheimer disease and related dementias (ADRD), including physician awareness of clinical trial opportunities, availability of study partners who can provide information on the study subject’s functioning, the invasiveness of procedures often performed in Alzheimer trials, and concerns about labeling a patient with a serious dementia diagnosis with no known treatment [2].

Accurate prediction of the future onset of ADRD has several important practical applications. In particular, it facilitates the identification of individuals who are at high risk of developing ADRD to support the clinical development of novel treatments. Commonly, patients are identified after they are already symptomatic and have already experienced significant neurodegeneration. Screening patients into high-risk groups can facilitate the development of programs that investigate causal relations to specific ADRD etiologies and recruitment to clinical trials. Persons predicted to be at risk can also be offered the opportunity to plan more thoughtfully for the future while retaining their cognitive function.

A number of previous dementia risk models have been published in peer-reviewed literature [2-10]. Most of these studies used clinical data for model estimation, which limits their generalizability to other settings. This paper extends previous research by basing model estimation on a very large integrated dataset of medical claims and electronic health record (EHR) data as well as the use of more sophisticated machine learning estimation methods than those used in most previous studies. The use of medical claims and EHR data facilitates the use of the model in settings where large numbers of patients are treated, resulting in the identification of much larger potential patient populations for clinical trial recruitment [3-12].

### Objectives

Nori et al [12] showed that machine learning models predict the onset of ADRD using medical claims and structured clinical data can have good performance near the time of onset and that performance diminishes with increasing time before onset. This study adds clinical notes data to those datasets to enhance the accuracy of the models and determines the prevalence of cognitive concerns in patient clinical notes up to 10 years before onset.

The quality of the clinical notes’ models depends on common semantics in electronic medical record (EMR) systems. In their groundbreaking work on using EMR data for machine learning, Rajkomar et al [13] admitted, “Our current approach does not harmonize data between sites,” but it can achieve similar accuracy at sites with sufficient volumes of data. Our study uses a dataset gathered from dozens of provider groups, mostly large integrated delivery network or hospital systems [14], and applies natural language processing (NLP) tools in a simple way to map semantically similar terms into concepts used in the models [15,16]. The processing of the clinical notes in this study favors automation, not clinical insight and expertise. This focus allows the methods to scale with little clinical intervention as new provider groups, and even new concepts are added to the data.

The use of a commercially available deidentified dataset will allow new studies to further refine the methods introduced here.

## Methods

### Overview

This study used deidentified medical claims and EHR data between 2007 and 2017 from the OptumLabs Data Warehouse (OLDW) [14]. The database contains longitudinal health information on enrollees and patients, representing a diverse mixture of ages, ethnicities, and geographical regions across the United States. The data in OLDW include medical and pharmacy claims, laboratory results, and enrollment records for commercial and Medicare Advantage enrollees. Clinical notes are available from a subset of EMR systems that chose to share these data. As this study involved analysis of preexisting, deidentified data, it was exempt from institutional review board approval [14].

### Data Sets

This study uses and extends the clinical dataset of Nori et al [12]. That work created a matched case-control cohort of patients with onset of ADRD (cases) and patients with no history of any ADRD (controls). Index dates vary from 2009 to 2017 with 2 years of data per patient. In that earlier work, 7 different models with lead times of 0, 3, 4, 5, 6, 7, and 8 years to index were created from structured EHR and medical claims data to understand how predictive accuracy can be sustained over time. These models are called structured models because they only use structured data—diagnosis codes, procedure codes, and prescriptions—from the EMR and medical claims systems.

The outcome variable in this study was a confirmed incident diagnosis of ADRD, which includes mild cognitive impairment and forms of dementia but not alcohol-induced dementia [12]. These multiple forms of dementia diagnoses were included in the outcome after consultation with clinicians, and a review of the data indicated that specific diagnoses of a single type of dementia are less reliable, and elderly patients often have multiple dementias at onset [10,11].

**Figure 1:**
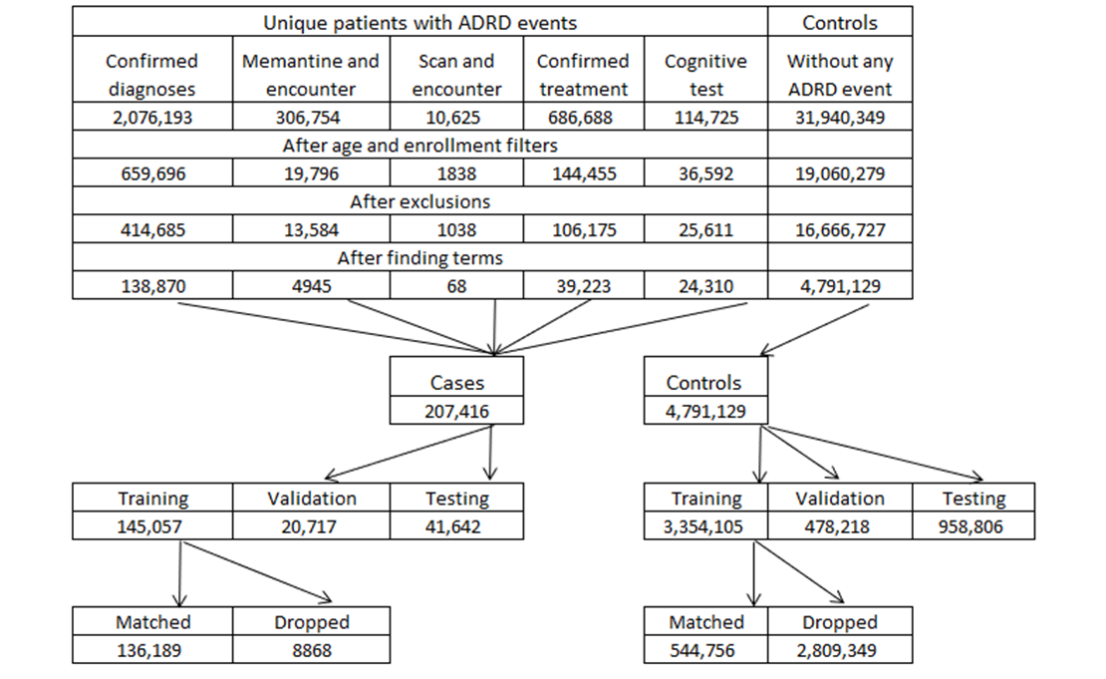
Attrition table. ADRD: Alzheimer disease and related dementias.

This study uses the clinical notes of the same patients from structured data. Not all EMR systems provide raw clinical notes to the data collection process, so clinical notes are available because of data use agreements. Hence, patients’ clinical notes data are missing due to legal agreements, not at random per patient and per encounter. To participate in the clinical notes’ models, a patient must have 2 unique dates with a clinical note at least 31 days apart in the 2-year data collection period. The numbers of patients that met this threshold are provided in Table 1. No other adjustment for missing data was made. The attrition table of the population is shown in Figure 1. The first 3 filters are the same as those of Nori et al’s study [12], with only the last filter of availability of clinical note data being specific to this analysis.

**Table 1:** Demographics of the study population.

Years to index date	Training set	N	Age, mean (SD)	Encounters, mean (SD)	Cases, n (%)	Females, n (%)
0	Matched training	680,945	74.3 (10.5)	30.5 (28.2)	136,189 (20.00)	417,390 (61.30)
3	Matched training	197,430	71.4 (10.2)	24.4 (21.9)	39,486 (20.00)	121,015 (61.30)
4	Matched training	130,270	70.4 (10.0)	22.5 (20.3)	26,054 (20.00)	79,795 (61.25)
5	Matched training	82,105	69.5 (9.8)	20.4 (18.8)	16,421 (20.00)	50,300 (61.26)
6	Matched training	47,555	68.6 (9.5)	18.3 (16.8)	9511 (20.00)	29,620 (62.29)
7	Matched training	23,455	67.6 (9.3)	16.6 (16.1)	4691 (20.00)	14,630 (62.37)
8	Matched training	7870	66.6 (9.1)	16.2 (15.8)	1574 (20.00)	4885 (62.07)
0	Validation	498,935	62.4 (11.3)	21.0 (21.8)	20,717 (4.15)	292,683 (58.66)
3	Validation	98,890	62.1 (10.8)	18.5 (17.9)	6525 (6.60)	60,560 (61.24)
4	Validation	61,471	61.7 (10.6)	17.4 (17.0)	4362 (7.10)	37,909 (61.67)
5	Validation	36,316	61.3 (10.4)	16.2 (15.9)	2763 (7.61)	22,464 (61.86)
6	Validation	18,888	61.0 (10.2)	15.2 (15.0)	1604 (8.49)	11,776 (62.35)
7	Validation	8186	60.6 (10.0)	14.2 (14.4)	806 (9.85)	5122 (62.57)
8	Validation	2660	60.0 (9.8)	14.0 (13.7)	292 (10.98)	1694 (63.68)
0	Test	1,000,448	62.3 (11.3)	21.0 (21.7)	41,642 (4.16)	587,326 (58.71)
3	Test	198,074	62.1 (10.8)	18.5 (18.0)	13,064 (6.60)	122,003 (61.59)
4	Test	122,939	61.7 (10.6)	17.4 (16.9)	8810 (7.17)	75,996 (61.82)
5	Test	72,304	61.3 (10.5)	16.1 (15.7)	5561 (7.69)	44,926 (62.13)
6	Test	37,938	61.0 (10.3)	14.9 (14.3)	3248 (8.56)	23,709 (62.49)
7	Test	16,487	60.8 (10.2)	14.1 (13.5)	1673 (10.15)	10,465 (63.47)
8	Test	5382	60.5 (10.1)	13.9 (13.8)	586 (10.89)	3474 (64.55)

Figure 1 shows that patients who entered the cohort via a scan and a confirmatory diagnosis rarely had clinical notes that met the thresholds for inclusion (68 of 1038 patients). The mean number of encounters per patient is 21, but the patients that enter by the scan rule have only 4.4 encounters. This low encounter count before filtering by clinical note days indicates that there is little opportunity to have 2 days with clinical notes separated by a month. It is likely that these patients have encounters in a specialty setting where there is no complete view of the patient’s health history.

As in Nori et al’s study [12], cases and controls were matched for age, gender, number of encounters, and index year at a 1:4 ratio to reduce confounding of variables. This step is important to improve interpretability of variables and reduce multicollinearity because of age, which, if not performed, would lead to erroneous importance of age-related variables [17-21]. Due to filtering by days with a clinical note, the cases must be matched to controls anew in this work versus reusing the same sampling as in Nori et al’s study [12].

### Clinical Notes

The raw clinical notes go through the Optum proprietary NLP for determination of all patients’ medical concept extraction. NLP concepts are identified and created based on broad topics such as medications, signs, disease and symptoms, measurements, and observations. The data are harvested from the clinical notes fields within the EMRs provided to Optum from over 50 large health care systems throughout the United States. The data used for the development of each NLP concept are deidentified, so the authors have no access to the raw notes.

The authors had access to the deidentified NLP data, which contains the date of the note, an occurrence date, a term, a sentiment, and possibly a family member. The terms are nouns, or abbreviations, extracted from the notes; sentiment describes the use of the noun (present, negative, possible, exhibit, exhibit.not, discuss, deny, concern, complain, etc). The content of the clinical notes are either about the patient or can be from a medical history where the content is about a family member. Family membership can be specific (mother, father, sibling, etc), vague (mother’s relations, ancestor, and boyfriend), or a combination of relationships. The occurrence date may differ from the note date if the original text makes a temporal statement such as “a year ago the patient complained of…” This study’s modeling uses the occurrence date of the term, if it exists, otherwise the date of the clinical note. We mapped the family members into 3 classes: immediate, family, and other (see Multimedia Appendix 1 for details). This mapping is based on wildcard word matching, so it is simple to implement, but may have errors. There are 29,528 unique terms and 1042 unique sentiments (42 positive sentiments such as “present,” “exhibit,” “observe”) with at least hundred patient clinical notes in all 7 yearly cohorts.

The NLP data have additional details, such as body location, severity, extent, and duration, that are not used in this study.

With the large number of unique sentiments (1042), the study decided to use only positive sentiment terms, indicating the presence of the term. In the raw notes, many negative terms are a collection of EMR survey questions *Patient denies smoking*, *Patient denies depression*, etc. These negative terms were excluded to reduce the complexity of processing the data and handling 1000 nonpositive sentiments.

Table 2 shows the highest 20 relative risk diagnoses in the onset year and their risks in the earlier years. The relative risk is the ratio of the probability of that diagnosis in cases versus controls. Each diagnosis must be supported by more than 10 cases, 10 controls, and 99 combined patients. Empty cells indicate that this threshold was not met. Years 7 and 8 had no unsuppressed values. These high-risk terms decay quickly over time; only 5 of the top 20 terms remain available for the model at year 3 and only 1 at year 4. Additional tables of terms, including tables with the most common terms, and the most common comorbidities are given in Multimedia Appendix 1.

**Table 2:** Top 20 relative risks of diagnosis.

Diagnosis	International Classification of Diseases, Ninth Revision code	Relative risk at years to index date				
		0	3	4	5	6
Wandering in diseases classified elsewhere	V403.1	21.57	—^a^	—	—	—
Unspecified senile psychotic condition	290.9	19.26	—	—	—	—
Unspecified persistent mental disorders due to conditions classified elsewhere	294.9	17.38	6.78	5.89	6.20	5.07
Senility without mention of psychosis	797.	16.48	—	—	—	—
Other general symptoms	780.9	16.27	—	—	—	—
Unspecified nonpsychotic mental disorder following organic brain damage	310.9	16.25	5.92	—	—	—
Other specified nonpsychotic mental disorders following organic brain damage	310.89	15.99	—	—	—	—
Other specified nonpsychotic mental disorder following organic brain damage	310.8	15.78	—	—	—	—
Other signs and symptoms involving cognition	799.59	15.06	4.45	—	—	—
Frontal lobe executive functional deficit	799.55	15.01	—	—	—	—
Dissociative amnesia	300.12	13.52	—	—	—	—
Personality change due to conditions classified elsewhere	310.1	13.52	4.42	—	—	—
Factitious disorder with predominantly psychological signs and symptoms	300.16	13.39	—	—	—	—
Psychotic disorder with delusions in conditions classified elsewhere	293.81	12.86	—	—	—	—
Confusional arousals	327.41	12.48	—	—	—	—
Visuospatial deficit	799.53	12.42	—	—	—	—
Reactive confusion	298.2	12.21	4.54	—	—	—
Subacute delirium	293.1	12.15	—	—	—	—
Alcohol-induced persisting amnestic disorder	291.1	12.07	—	—	—	—
Frontal lobe syndrome	310.0	12.07	—	—	—	—

^a^Not applicable.

### Clinical Notes Clusters

With 29,528 unique terms in all the datasets, and without access to the algorithms that create the terms, the study needed to determine how the terms map to clinical concepts. In the raw clinical note, we expect that different clinicians will have alternative spellings—mi, ami, or acute myocardial infarction; htn or hypertension—depending on their training, the EMR they use, and many other factors. This study’s upstream NLP does not map these terms into concepts but leaves them in their raw form. This creates a need to gather alternative spellings and related clinical terms into groups, or clusters, before using them. Without such a grouping, an individual term’s impact may be diluted to the point of uselessness due to idiosyncratic abbreviations, spellings, and synonyms (eg, Alzheimer disease vs Alzheimer dementia). The methods here will ameliorate this situation. The terms are filtered to terms having at least 500 patients in any annual model, yielding 14,236 terms.

It would be possible, but time consuming, to map these terms to a medical ontology, but the study decided to pursue an algorithmic strategy relying on additional NLP processing. The end goal of this step is to map the terms to data-driven concepts that will group similar terms into more powerful machine learning features. The positive sentiment patient terms are processed into a sequence of terms, ordered by date, for each patient. Most of the clinical notes data lack a specific time of day, so the terms have no order other than a date. If the raw NLP provided a sequence number for the extracted terms, then that sequence could be used to order the patient’s terms. Term sequences with less than 50 characters long are omitted. Due to database limits on the length of a single character field, the process needed to count characters in the concatenated terms. The choice was made to use these character counts to limit the patient stories used. In total, 50 characters is approximately 8 distinct terms. The word windows used are 10 words long.

At this step, there is a term-based *story* of 50-31,341 terms for each patient. The story file for all patients across all years is 5.9 gigabytes of text. The training text is collected and analyzed as 1 text file, with a row for each patient containing the patient’s terms. To limit duplication of data from overlapping model years, model years 4 and 6 are not in the NLP training model (all year 4 terms are in either year 3 or year 5).

This text file can be analyzed using any NLP algorithm to build semantic knowledge among its words. The study chose to use Fasttext by the Facebook artificial intelligence research team for its speed and simplicity [22]. Fasttext builds a conditional probability model of term appearances in the context of their surrounding terms. The output of Fasttext is a numeric vector for each term. These term vectors are a meaningful mapping of each term to a vector space where the vector distance maintains word similarity. Thus, if 2 terms are very close to each other, measured by their vector distance, then they are synonyms. Alternate spellings of the same medical concept should be nearby in the vector space because the terms that surround them in the clinical notes will be similar. The study chose 100-dimension vectors to embed the terms.

To run the Fasttext algorithm, we chose the unsupervised continuous bag-of-words option with 8 epochs and a window of 10 terms. We explicitly turn off subwords because we do not want the algorithm associating the term alzheimers_disease with crohns_disease based on common subwords (syndrome is another confusing subword). Our reliance on terms from an upstream process means that misspellings are not an issue in this context. The unsupervised option means that Fasttext is finding semantic relations among the terms. The word window of 10 terms limits the probability model to overlapping sequences of 10 terms. The lack of sequence information on terms within a day means that the method needs more data to obtain a more accurate probability model of the text relations. Fasttext returns 11,061 terms due to its own filtering.

Once the study has the vector mapping of each term from Fasttext, the terms can be clustered into similar groups using the Euclidean distance of the terms as the similarity measure [23]. This is performed with the hclust function in R v3.5.1 [24]. As the goal of this clustering is to create features for the predictive model, we chose a large number of clusters, 1106 or 10% of the terms. A manual inspection of the clusters indicated that a much larger number of clusters (2212 or 5%) may split important sets of terms, and fewer clusters would merge groups that are less related to the outcome. Multimedia Appendix 1 shows the clusters for terms with individual memory and cognition terms. For example, the terms memory_loss, memory_issues, forgetful, memory, mild_cognitive_impairment, mci, recalling_issues, lewy_body_dementia, pseudodementia, memory_dysfunction, frontotemporal_dementia, and short_term_memory_loss all group together with a few more terms in 1 cluster. No manual editing of the clusters was performed. Note that the use of the embedding on training terms across all years, and the clustering of only those terms effectively omits novel terms present in the test data. However, because terms are the result of the upstream NLP over all years, the introduction of new terms in testing is rare. In production, new terms can be mapped into clusters if necessary.

The models use all the same medical features as the structured models and add 2 sets of features for the terms. One set of features counts the unique days with a specific term attributed to an individual or 1 of the 3 family types. The other set of features counts unique days at the clustered term level; thus, every term may appear twice, once specifically and once in its cluster.

### Machine Learning

After computing these features, the same feature filtering method proposed by Nori et al [12] is applied. These filters remove features without sufficient support in the data as well as features whose ratio of matched to unmatched odds ratios are too extreme (see Multimedia Appendix 1). The filter based on the ratio of matched to unmatched odds avoids the inclusion of terms that are primarily associated with age and not with the outcome. Age-related terms can have a high unmatched odds ratio but a low matched odds ratio; thus, the ratio of these 2 values can filter these outliers. The ratio thresholds of 0.5 and 4 are used to remove features that are too skewed to age. For example, screening mammography has a very low unmatched odds ratio because it skews highly to younger women; the ratio of its unmatched to matched odds would be less than 0.5, and it would be removed from the model.

The models are fit with LightGBM [25], an algorithm that fits a gradient boosting machine to the 0 or 1 outcome variable using a series of decision trees. After solving the first model with a tree, another model is fit with a new outcome initialized to the residuals of the prior tree’s fit. The series of residual fits optimize the loss function of the original model [26,27]. LightGBM also uses advanced methods such as sampling the feature space and pooling features into importance sets for improved performance. This study varied the parameters of LightGBM using a grid search; the model quality is assessed on the validation data, and the best model is selected. The study searched the feature fractions (.25, .2, .15), learning rates (.015, .01, .02), minimum data in leaf (1000, 800, 500), number of trees (300), and size of trees (127, 63). Each model was allowed to search independently for the parameter set that maximized its quality, as measured by the validation set’s positive predictive value (PPV).

## Results

This study reports 3 summary measures of model quality. The area under the curve (AUC) score is the probability that a random case score is higher than a random control. A drawback of AUC is that it is a global measure of discrimination, and it does not reflect the decision boundary to take action on a score from the model. With this in mind, we report 2 other measures, the PPV and lift.

PPV is the percentage of true positives in the at-risk population. To compute the PPV across this population, where the prevalence varies widely by age, we apply a threshold per age group where the number of patients at risk in each age group matches the age-based prevalence of cases.

Lift is the ratio of PPV to prevalence. Its values range from 0 to infinity. The lift reflects the improvement in the model over a random choice. For a rare disease, PPV may be low because the outcome is hard to detect and dividing by the prevalence provides a standardized way to correct for the prevalence.

**Figure 2:**
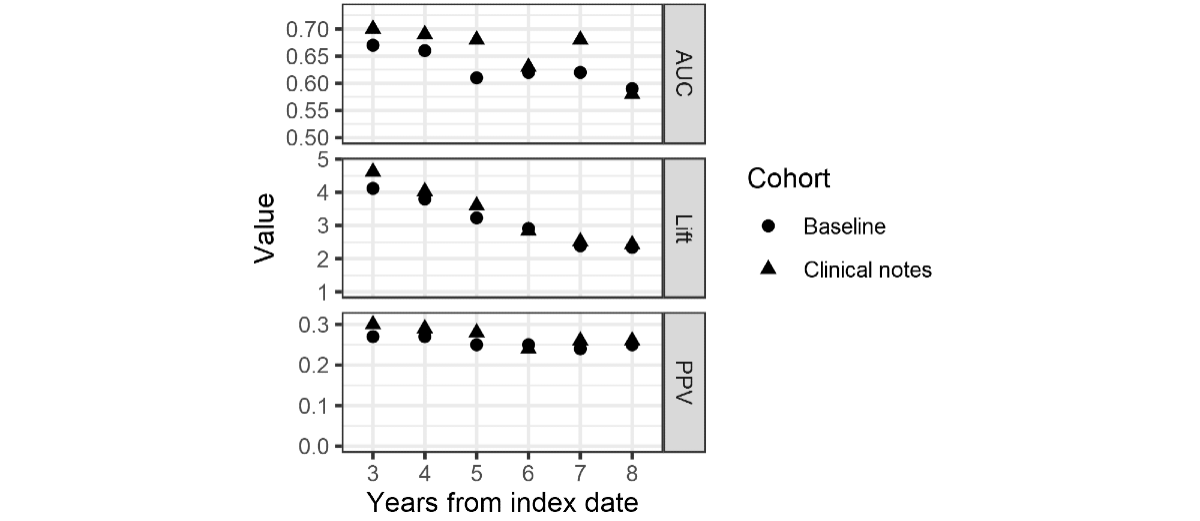
Model quality measures. AUC: area under the curve; PPV: positive predictive value.

Table 3 and Figure 2 show the model quality statistics for the baseline models without the clinical notes features and after adding the notes’ terms and term clusters. In general, the models with clinical notes always have a higher AUC score, and the PPV and lift scores are higher in all but 1 year. Averaged over all models, the PPV was 5 points higher and the AUC was 4 points higher when terms and term clusters were included. Year 0 baseline values were reported by Nori et al [12].

**Table 3:** Quality of model fit on the test data.

Year	Sensitivity		Specificity		Area under the curve		Lift	
	Baseline	Clinical notes	Baseline	Clinical notes	Baseline	Clinical notes	Baseline	Clinical notes
0	0.45	0.68	0.98	0.99	0.84	0.94	13.92	16.39
3	0.27	0.30	0.95	0.95	0.67	0.70	4.12	4.62
4	0.27	0.29	0.94	0.95	0.66	0.69	3.80	4.03
5	0.25	0.28	0.94	0.94	0.61	0.68	3.23	3.60
6	0.25	0.24	0.93	0.93	0.62	0.63	2.91	2.84
7	0.24	0.26	0.91	0.92	0.62	0.68	2.39	2.52
8	0.25	0.26	0.91	0.91	0.59	0.58	2.34	2.43

Table 4 shows the most important features in the onset year. The variable naming convention is *cls*, which is the prefix for cluster variables; *idv* for individual terms; *OFam* for other family; *IFam* for immediate family; *ETG* for episode treatment groups; *RXG* for medication therapeutic groups; *ICD* for International Classification of Disease version 9 (ICD-9); and *CPT* for Current Procedures and Terminology version 4. Tree-based machine learning algorithms rank variables by gain—how much the fit improves after that feature is used in a tree node. The gain is a dimensionless value, so we report the percentage of total gain attributed to each feature for all features up to 80% of the total gain. We refer to variables that meet this threshold as *important* variables. Important variables for the other years are shown in Multimedia Appendix 1.

**Table 4:** Important variables at onset (year 0) Total Gain (N) is 22,040,569.

Variable type	Variable name	Gain, n	Percent gain	Cumulative percent gain
cls	Dementia and Alzheimer dementia	3,298,549	15.0	15.0
cls	Memory loss and memory issues	2,833,536	12.9	27.8
idv	Dementia	2,162,843	9.8	37.6
idv	memory_issues	1,525,697	6.9	44.6
idv	memory_loss	1,498,113	6.8	51.4
idv	mild_cognitive_impairment	459,131	2.1	53.4
idv	Forgetful	419,780	1.9	55.3
cls	Alzheimer disease and other family memory issues	382,811	1.7	57.1
ETG	Neurological diseases signs and symptoms	378,955	1.7	58.8
idv	cognitive_impairment	346,991	1.6	60.4
idv	Memory	337,701	1.5	61.9
ICD	Altered mental status	275,533	1.3	63.2
idv	memory_lapses	256,076	1.2	64.3
idv	short_term_memory_loss	252,683	1.1	65.5
CPT	Neuropsychological testing (eg, Halstead-Reitan neuropsychological battery, Wechsler memory scales, and Wisconsin card sorting test), per hour of the psychologist’s or physician’s time, both face-to-face time administering tests to the patient and time interpreting these test results and preparing the report	245,279	1.1	66.6
cls	Cognitive impairment and hearing impairment	232,700	1.1	67.6
idv	Alzheimers_disease	221,324	1.0	68.6
cls	Cognitive issues and cognitive disorder	214,553	1.0	69.6
ETG	Mood disorder, depressed	214,171	1.0	70.6
CPT	Magnetic resonance (eg, proton) imaging, brain (including brain stem); without contrast material	213,180	1.0	71.5
ICD	Unspecified persistent mental disorders due to conditions classified elsewhere	174,643	0.8	72.3
cls	Memory lapses and concentrating	163,176	0.7	73.1
idv	getting_lost	159,014	0.7	73.8
CPT	Computed tomography, head or brain; without contrast material	150,658	0.7	74.5
cls	Family dementia and memory disturbance	125,350	0.6	75.1
ETG	Psychotic and schizophrenic disorders	121,595	0.6	75.6
dem	Age	118,598	0.5	76.1
RXG	Atypical antipsychotics	115,677	0.5	76.7
ETG	Mental disorders, organic and drug-induced	114,621	0.5	77.2
cls	Pain and tenderness	106,109	0.5	77.7
CPT	Neuropsychological testing (eg, Halstead-Reitan neuropsychological battery, Wechsler memory scales, and Wisconsin card sorting test), with qualified health care professional interpretation and report, administered by technician, per hour of technician time, face-to-face	100,425	0.5	78.1
dem	Number of encounters	94,671	0.4	78.6
RXG	Selective serotonin reuptake inhibitors	93,374	0.4	79.0
OFam	informant	92,567	0.4	79.4
idv	relaxing_issues	84,741	0.4	79.8
ICD	Depressive disorder, not elsewhere classified	77,831	0.4	80.1

**Figure 3:**
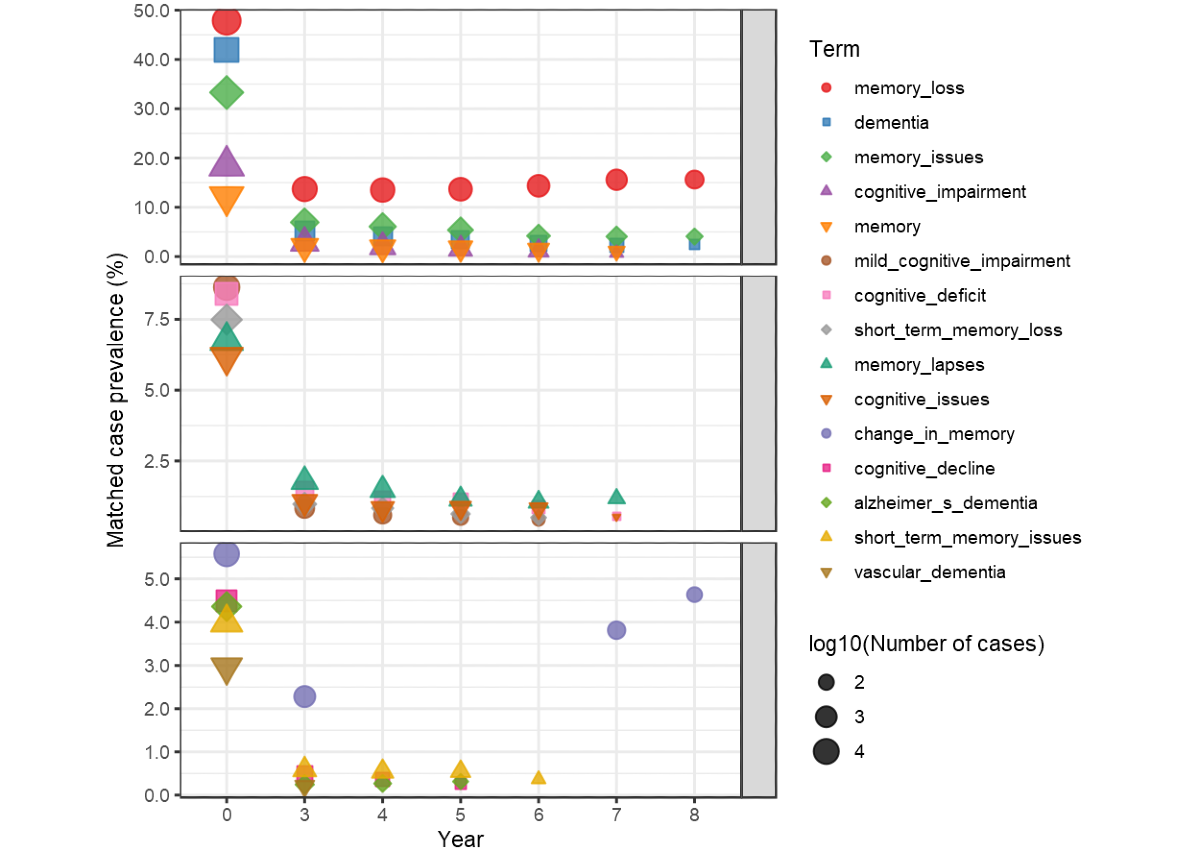
Frequency of cognitive terms in cases.

**Figure 4:**
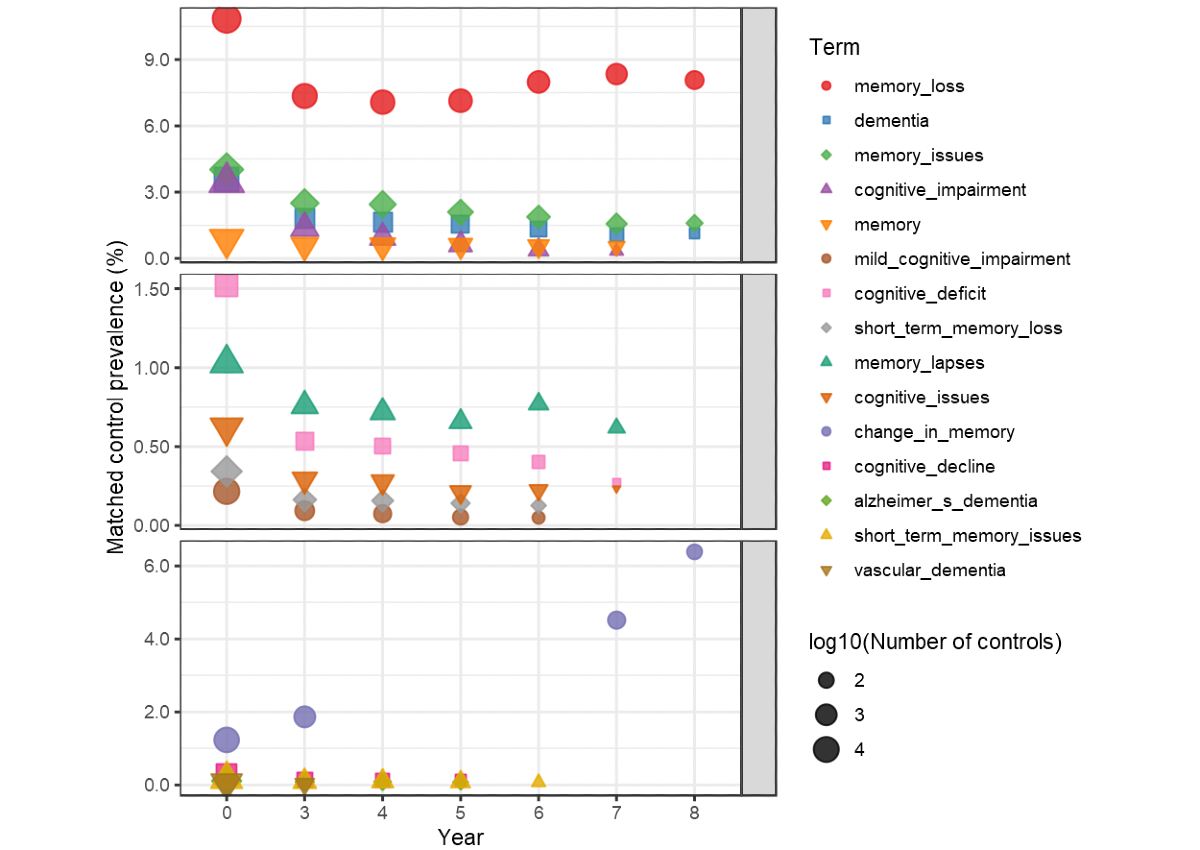
Frequency of cognitive terms in controls.

Figures 3 and 4 extract terms that contain the phrases memory, dementia, and root *cognit* to see how the terms’ prevalence varies over time. The top 15 terms ordered by matched case prevalence are displayed in 3 groups of 5 to allow scaling of the *y*-axis in each group. The plots show the prevalence of the unmatched validation data. The terms are present in each year if they are part of the model features for that year, but the filtering rules can omit them. For example, mild_cognitive_impairment appears in model years 0 through 6 but not in years 7 and 8 due to filtering. The cluster containing mild_cognitive_impairment is in all models and is important in all but the year 8 model. Figure 4 shows the plot of these terms in the control population, that is, a positive term for those without an incident diagnosis.

## Discussion

### Principal Findings

It is remarkable that even with all the diagnosis and prescription codes as well as neurological testing and radiology procedures available from the claims and structured EMR data, the clinical notes terms account for the first 8 and 21 of the 36 top predictors in the onset year model (Table 4). This indicates that the EHR data collection process collects important terms and that the NLP workflow is processing the clinical notes in a helpful manner. The most important non-note features are mood disorders, especially depression, psychoses, and prescription treatments for those disorders. It also may indicate that the clinical notes terms are a better indication of the prognostic symptoms than the structured data.

In the longer-term predictions, there are 3 data factors involved in decreasing accuracy. First, the cohorts rapidly decrease in size; for example, the training data at year 8 is 1% the size of the onset year (Table 1). This decrease in size is not just a survivorship issue, but the clinical notes data collection process was in its first year in year 8, so the diminishing size is a reflection of data collection growth from year 8 to year 0. Finally, there is commensurate growth in the features present in the model from 3450 to 7391 from year 8 to year 0, including all the medical coded features. As the scope of the data asset grows, it is possible that the future version of this model could perform better with little new modeling effort.

With ample evidence that ADRD is under coded [28-31], this dataset shows the existence of clinical notes with positive sentiment of many terms related to ADRD many years preceding the onset date of the cohort. The existence and ability of the model to extract terms such as memory loss, agitation, anxiety, and depression in the year 6 model (see Multimedia Appendix 1) demonstrates that these concerns are being coded in the clinical notes well before the ADRD diagnosis is recorded.

The family history terms are not as helpful as the individual terms. The only family term that survives in the important predictors is an immediate family history of Alzheimer disease only in the models for years 3, 4, and 5, but never over 0.3% of the total gain.

Figures 3 and 4 indicate that memory loss and other terms involving cognition and dementia are present at higher rates than one may expect. Memory loss is present in more than 13.52% (3522/26,054) of cases throughout the model years. Clearly, one wonders if the cases with these terms have a delayed diagnosis in the structural data. Furthermore, Figure 4 shows that the controls had at least 7.07% (7364/104,216) prevalence of memory loss in all model years. Although not all memory loss is an indication of ADRD, these prevalences are in a population whose mean age is in the low 60’s (Table 1) and could be an indication of under coding ADRD in the controls. However, the crux of the issue is that these memory terms in clinical notes may not reflect the underlying physiological changes of dementia or under coding.

### Comparison With Previous Work

The structured models fit by Nori et al [12] found evidence of increased mental health, neurological testing, and anticholinergic risk factors found in other studies, as well as cardiovascular risk, which has been associated with vascular dementia [31-33]. The structured models in Nori et al’s study [12] did not confirm diabetes mellitus as a risk factor, as found in Haan’s study [33]. However, this study does find diagnoses of diabetes mellitus as important in the 3-, 4-, 5-, and 8-year models both as a coded diagnosis as well as in the notes. In addition, the clinical notes as far back as 6 years do identify metabolic syndrome and a cluster of terms related to insulin resistance as important (see Multimedia Appendix 1). This is important because there is an ICD code for metabolic syndrome, but that code does not surface in the structured data models. This provides some evidence in support of Haan’s study [33], which is not present in the structured model.

Several previous studies have attempted to model the risk of Alzheimer and related disorders. Most of these have been small studies using detailed clinical data [3-9]. Recently, several studies have used widely available claims data to predict the onset of Alzheimer and related disorders [10-12]. These claims-based models achieved similar results similar to those of earlier studies (AUC ranging from 0.60 to 0.78). Our study shows that incorporating EHR data into the analysis results in significant additional improvements in the performance of models predicting Alzheimer and related disorders.

### Limitations

This analysis was conducted using administrative claims and EHR data. The accuracy of diagnostic coding is a known issue with claims data. In the case of dementia, diagnostic inaccuracy is especially challenging in distinguishing between its different forms. In related work, we are exploring methods that explicitly address errors in the labeling of those who have dementia, and this appears to be promising [12]. Nevertheless, it is interesting to note that, despite the diagnostic coding issues, our models perform on par (or better) than previously published models using much deeper clinical information.

### Conclusions

Our findings have important implications for the usefulness of predictive models based on administrative claims and EMR data to identify individuals at risk of dementia. Given the widespread availability of claims data that are already routinely used to identify individuals for interventions such as disease management programs, it is clear that predictive models could clearly be much more widely used to support individuals at risk of dementia in the community to help delay or even prevent institutionalization in nursing homes as well as aid in financial planning and provide other support needed by families having a member with dementia.

Similarly, the widespread availability of EHRs in clinical settings would enable clinicians to make use of predictive models to support their patients with dementia and their families. In the rarer settings where both claims and EHR data are available, our findings indicate that predictive models will be even more effective at identifying patients with dementia who could benefit from social support.

A second, very important, application of machine learning models is to identify patients for recruitment into clinical trials. Collecting the clinical data needed for screening dementia patients for clinical trials is extremely expensive—in the case of Alzheimer disease estimated to be over US $4000 to screen patients with cognitive assessments and positron emission tomography scans [34,35]. This cost is inflated by the need to screen many individuals for every individual identified. Any tool that reduces the number of patients needing to be screened will reduce the cost of patient recruitment. As reported in Table 3, models using EHR data correctly identified 2.5 times the number of patients with dementia relative to baseline prevalence 8 years to diagnosis and 16.4 times the baseline prevalence at the time of diagnosis. Although model performance declines further from diagnosis, these results suggest that predictive models based on machine learning methods could also be helpful in identifying patients earlier in their disease course. This is important for both the provision of social support and clinical trial recruitment. In the case of social support, it may well be the case that intervening earlier will be more effective in delaying nursing home institutionalization, and it would certainly give families more time to prepare for such an outcome. In the case of clinical trials, it is possible that recruiting patients who are earlier in the disease course may improve the effectiveness of pharmacologic interventions, which, to date, have been of little clinical value.

Clinical notes data extracted into deidentified structured tables can be useful in adding value to models built with structured data. The accuracy of the onset year model is much higher than that of other models in the literature (94% AUC vs in the 70% range) [8,20-33]. This ability to discriminate with a PPV of 68% (51% higher than without notes) means that this model can be an effective screening tool for patient and provider follow-up. The rapid decline in model quality beyond diagnosis limits the utility of the model for long-term prediction. It is unclear if this decline can be easily remedied once more clinical notes data are available or if this indicates a more important issue of primary data collection and under coding with more challenging remedies.

Clustering terms from the deidentified clinical notes helps to overcome variation in how clinical notes are written across diverse provider groups. The term clusters also boost the strength of the group by combining similar concepts into a coherent feature that improves prediction.

Future work should focus on semisupervised approaches that expand the data available for training by learning to label data in a consistent manner. Work on semisupervised methods can also help enhance the reliability of case/control labeling for ADRD, as started by Nori et al [12].

Recent work by Xie [36] shows promise for augmenting labeled data with use cases outside the medical domain. It is not yet clear if these methods can augment/perturb medical record data in a way that can boost model performance.

## Appendix

Multimedia Appendix 1 Project analytical overview and additional results.

## Abbreviations

ADRD: Alzheimer disease and related dementias
AUC: area under the curve
EHR: electronic health record
EMR: electronic medical record
ICD: International Classification of Disease
NLP: natural language processing
OLDW: OptumLabs Data Warehouse
PPV: positive predictive value
