# Supplementary material for: Predicting Onset of Dementia Using Clinical Notes and Machine Learning: Case-Control Study
Source: JMIR Med Inform. 2020 Jun 3;8(6):e17819. doi: 10.2196/17819 (PMC7301255; doi:10.2196/17819)
Supplement: Multimedia Appendix 1 [file medinform_v8i6e17819_app1.docx]

Appendix

Contents

[Project Analytical Overview 2](#_Toc32909300)

[Family Terms 3](#_Toc32909301)

[Sentiment 5](#_Toc32909302)

[Top 20 ICD-9 Diagnosis codes in Cohort in Index Year 6](#_Toc32909303)

[Top 20 Terms by year and patient days 7](#_Toc32909304)

[Top 20 Immediate Family Terms 8](#_Toc32909305)

[Top 20 Terms like ‘memory’ 9](#_Toc32909306)

[Clusters 10](#_Toc32909307)

[Confusion 10](#_Toc32909308)

[Memory Loss 12](#_Toc32909309)

[Change in Memory 12](#_Toc32909310)

[Cognitive Impairment 13](#_Toc32909311)

[Alzheimer’s Disease 13](#_Toc32909312)

[Alertness and Cognitive Deficit 13](#_Toc32909313)

[Memory Lapses 13](#_Toc32909314)

[Cognitive Issues 15](#_Toc32909315)

[Family History of dementia 15](#_Toc32909316)

[Clumsy Cognition Issues 15](#_Toc32909317)

[Cognition impaired 15](#_Toc32909318)

[Memory Changes 16](#_Toc32909319)

[Memory Abnormality 17](#_Toc32909320)

[Odds filtering 17](#_Toc32909321)

[Important Variables in Each model year 17](#_Toc32909322)

[Year 3 17](#_Toc32909323)

[Year 4 19](#_Toc32909324)

[Year 5 22](#_Toc32909325)

[Year 6 24](#_Toc32909326)

[Year 7 25](#_Toc32909327)

[Year 8 26](#_Toc32909328)

# Project Analytical Overview

The project steps outline how a large company used clinical notes from an EHR to enhance a complex machine learning task. Government policy and company practice, along with use of proprietary software constrains the type of data available and the processing that can be done. Working with these limits is part of the daily process of many practitioners of machine learning. We hope our experience adds to the knowledge base and success of their projects.

Due to privacy reasons the project has no access to the raw NLP and only receives de-identified data with terms, sentiment, severity, duration, body part, family member and a date. This removes much flexibility from the analysis, but also simplifies it. The NLP output has no miss-spelled terms and there are no terms in the test data not found in the training. However, there also is no sentence structure remaining. The project only used the terms and sentiment fields of the data as the other fields were too specific for the project goals.

A typical encounter may have note terms and sentiments like this:


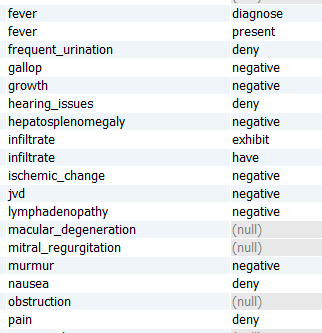


An encounter that investigates dementia may have terms like:


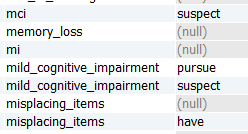


The (null) sentiments indicate the NLP processing was unable to assign a sentiment to the term, but the term was found in the note. Because the sentiment is undetermined, these terms are not used.

Terms are also very specific: ‘confused’, ‘confusion’, ‘confusional_state’, ‘confusional_episode’ and ‘left_right_confusion’ are all separate terms in the data. This means that specific terms may be too sparse to improve the modeling and they may not generalize well. This generalization is specifically important for generalization across hospital systems and their own medical dialects. The terms should be aggregated into higher level concepts before modeling. The aggregated concepts can then be used as features in the machine learning classifiers. This is achieved in the clustering step.

Processing outline:

1. Receive the pre-processed NLP terms and sentiments from the upstream NLP engine.
2. Map raw sentiments to ternary levels (positive, negative, and other). Keep only positive sentiments.
3. Using the terms from many years and many lead times, create a single text file for training an embedding of the terms using an unsupervised model. For FastText, each line in the file contains one patient’s terms in date order.
4. Using the embedding term vectors, cluster the terms to aggregate similar terms together. This yields a mapping of terms to their cluster id.
5. Augment the original machine learning model features with the cluster ids of the terms found in each patient’s notes.
6. Fit the new augmented model.

As with many large projects, a de-coupled workflow like this one (NLP in one team, embedding development with another team, etc.) has advantages of personnel assignment and managing workloads. This comes at the expense of additional accuracy of a tightly coupled process, or even a deep learning one where a feedback loop could link all the steps together.

In our case, the creation of the NLP terms resides in a large product team that serves hundreds of customers. Updating their engines for any one project is not possible without explicit consideration of the product impacts to all those customers.

# Family Terms

There are 832,740 distinct family descriptors in the data set. This large number arises from the unique combinations of family members recorded, e.g. “father and paternal grandfather and maternal uncle”, or “1-ST and 2-ND degree relatives”. The descriptor may also reference significant others and caregivers. The raw NLP also provides extra descriptors in the family text like the age of the person referenced that complicate the data processing. After filtering to family descriptors that appear at least 500 times there remain 5,455 unique descriptors. Due to these counts the study pursued a simplified assignment of family descriptors to 3 classes, Immediate, Family and Other.

Immediate family members are family descriptions that contain the words ‘mother’, ‘father’, ‘brother ’, ‘sister’, ‘sibling’ or ‘parent’. These words are wildcard matched within the description, so ‘grandmother’ will match ‘mother’ in Immediate Family but ‘maternal aunt’ will not. Family matches occurrences of the word ‘family’ and Other is the remainder. These matches are applied in order.

The goal here is not a precise assignment but a useful one for screening features into the model. Certainly more work could be done here to add precision to the labeling of the notes. The reason there are so many family member texts is that the description is an ordered set of all members mentioned, e.g. “mother, brother, aunt, uncle, ancestor, paternal grandfather, boyfriend” can be a unique text descriptor.

| **Family Type** | **Distinct family member text descriptors** |
| --- | --- |
| Immediate | 425,274 |
| Other | 337,976 |
| Family | 69,490 |

Despite the large number of family descriptors there are relatively few terms recorded for the family members.

| Number of NLP terms with at least 500 people prior to cohort building | |
| --- | --- |
| **Type of term** | **Unique positive terms** |
| Family | 374 |
| Immediate Family | 592 |
| Other Family | 646 |
| Individual | 7,498 |

# Sentiment

The following sentiments were assigned to positive and negative classes. Sentiments with unknown attribution to these classes and their terms are omitted. For some sentiments, it is unclear if the raw text would show an intent or an outcome, i.e. ‘diagnose’ or ‘diagnose.not’ but the lack of a sentiment with a past tense allowed the assignment to positive/negative groups.

| **Negative Sentiments** | **Positive Sentiments** | |
| --- | --- | --- |
| able.not | accept | feel |
| affected.not | active;recurrent | have |
| allergy.free | afflicted | have to |
| allergy-deny | allergy | infectious;disease |
| allergy-express.not | allergy-according to | intermittent;progression |
| allergy-have.not | allergy-complain | negative.not |
| allergy-negative | allergy-diagnose | observe |
| complain.not | allergy-exposure | positive |
| concern.not | allergy-general | potential |
| deny | allergy-have | present |
| diagnose.not | allergy-observe | primary;disease |
| exhibit.not | allergy-positive | progression |
| express.not | allergy-prn | re-admission |
| feel.not | chronic;disease | response |
| free | clinical;progression | risk |
| free-free | complain | serious;disease |
| have.not | constant;progression | stable;progressing |
| negative | diagnose |  |
| observe.not | diagnose + treat |  |
| pertinent_negatives | diagnosis/history |  |
| positive.not | disease |  |
| present.not | disease;successful |  |
| response;no | exhibit |  |
| seem.not | express |  |

The terms used in modeling are only positive sentiment terms.

# Top 20 ICD-9 Diagnosis codes in Cohort in Index Year

These comorbidities and medical events as coded by ICD-9 diagnosis codes are in the top 20 by index year prevalence, and may not be the most prevalent in the earlier years. Prevalence is in the global population, not training data.

| **ICD-9 Diagnosis Code** | | **Prevalence at Years to Index Date** | | | | | | |
| --- | --- | --- | --- | --- | --- | --- | --- | --- |
|  |  | **0** | **3** | **4** | **5** | **6** | **7** | **8** |
| Unspecified Essential Hypertension | 401.9 | 49% | 43% | 41% | 39% | 37% | 37% | 35% |
| Routine General Medical Examination At Health Care Facility | V70.0 | 45% | 47% | 45% | 43% | 41% | 37% | 32% |
| Other And Unspecified Hyperlipidemia | 272.4 | 42% | 44% | 43% | 41% | 40% | 39% | 38% |
| Other Screening Mammogram | V76.12 | 27% | 29% | 29% | 28% | 28% | 27% | 25% |
| Encounter For Long-Term (Current) Use Of Other Medications | V58.69 | 21% | 18% | 17% | 16% | 13% | 11% | 9% |
| Esophageal Reflux | 530.81 | 21% | 20% | 18% | 17% | 16% | 15% | 14% |
| Need For Prophylactic Vaccination And Inoculation Influenza | V04.81 | 20% | 29% | 29% | 29% | 29% | 29% | 27% |
| Need For Prophylactic Vaccination And Inoculation Against Viral Hepatitis | V05.3 | 20% | 7% | 6% | 5% | 4% | 4% | 3% |
| Diabetes Mellitus Without Mention Of Complication, Type Ii Or Unspecified Type, Not Stated As Uncontrolled | 250.00 | 19% | 19% | 18% | 17% | 16% | 16% | 15% |
| Pain In Soft Tissues Of Limb | 729.5 | 18% | 16% | 15% | 15% | 14% | 13% | 13% |
| Essential Hypertension, Benign | 401.1 | 17% | 23% | 23% | 23% | 22% | 19% | 17% |
| Special Screening For Malignant Neoplasms, Colon | V76.51 | 16% | 14% | 13% | 12% | 12% | 12% | 12% |
| Other Malaise And Fatigue | 780.79 | 16% | 15% | 14% | 13% | 13% | 12% | 12% |
| Lumbago | 724.2 | 16% | 13% | 12% | 12% | 11% | 11% | 10% |
| Pure Hypercholesterolemia | 272.0 | 15% | 18% | 18% | 18% | 17% | 16% | 14% |
| Chest Pain, Unspecified | 786.50 | 15% | 13% | 12% | 12% | 12% | 11% | 11% |
| Cough | 786.2 | 15% | 13% | 12% | 11% | 11% | 10% | 10% |
| Unspecified Hypothyroidism | 244.9 | 14% | 15% | 14% | 14% | 13% | 13% | 12% |
| Routine Gynecological Examination | V72.31 | 14% | 17% | 17% | 17% | 17% | 15% | 13% |
| Obesity, Unspecified | 278.00 | 14% | 11% | 10% | 9% | 9% | 9% | 9% |

# Top 20 Terms by year and patient days

These are the terms and counts in the unsupervised embedding models. Years 4 and 6 are intentionally omitted as indicated in the manuscript.

The “idv” prefix indicates this is an individual term, “Ifam” is immediate Family, “fFam” is family and “OFam” is other family.

| Patient Days with term | Index Year | | | | |  |
| --- | --- | --- | --- | --- | --- | --- |
| Term | 0 | 3 | 5 | 7 | 8 | Grand Total |
| idv_pain | 6,647,061 | 1,532,438 | 615,543 | 163,665 | 53,307 | 9,012,014 |
| idv_edema | 3,405,722 | 737,172 | 286,982 | 73,997 | 24,385 | 4,528,258 |
| idv_hypertension | 3,140,808 | 667,677 | 252,070 | 61,999 | 19,427 | 4,141,981 |
| idv_shortness_of_breath | 2,894,599 | 560,283 | 203,874 | 47,568 | 15,294 | 3,721,618 |
| idv_murmur | 2,662,806 | 638,702 | 263,168 | 72,123 | 23,732 | 3,660,531 |
| idv_allergies | 2,590,697 | 551,535 | 219,358 | 58,926 | 20,749 | 3,441,265 |
| idv_cough | 2,529,290 | 529,041 | 203,997 | 53,619 | 16,815 | 3,332,762 |
| idv_fever | 2,437,776 | 515,305 | 203,575 | 56,379 | 17,991 | 3,231,026 |
| idv_tenderness | 2,207,101 | 508,100 | 208,173 | 59,319 | 19,361 | 3,002,054 |
| idv_hyperlipidemia | 2,198,756 | 498,420 | 191,868 | 48,465 | 15,087 | 2,952,596 |
| idv_nausea | 2,198,841 | 449,842 | 171,307 | 42,933 | 12,952 | 2,875,875 |
| idv_diabetes | 2,142,492 | 481,549 | 182,664 | 47,792 | 15,854 | 2,870,351 |
| idv_vomiting | 2,000,905 | 392,433 | 150,069 | 38,618 | 11,673 | 2,593,698 |
| idv_gallop | 1,779,557 | 419,879 | 174,853 | 50,172 | 17,124 | 2,441,585 |
| idv_rub | 1,714,852 | 389,886 | 158,575 | 44,341 | 15,002 | 2,322,656 |
| idv_rash | 1,723,301 | 362,373 | 135,150 | 33,094 | 9,464 | 2,263,382 |
| idv_palpitations | 1,717,299 | 356,556 | 137,224 | 35,979 | 11,105 | 2,258,163 |
| idv_swelling | 1,699,130 | 363,101 | 138,027 | 35,063 | 11,134 | 2,246,455 |
| idv_headache | 1,697,349 | 341,182 | 128,018 | 32,012 | 9,745 | 2,208,306 |
| idv_lesion | 1,607,642 | 382,575 | 156,042 | 43,518 | 14,606 | 2,204,383 |

# Top 20 Immediate Family Terms

| Patient Days with Term | Index Year | | | | |  |
| --- | --- | --- | --- | --- | --- | --- |
| Term | 0 | 3 | 5 | 7 | 8 | Grand Total |
| Ifam_cancer | 634,652 | 127,520 | 52,176 | 14,584 | 4,282 | 833,214 |
| Ifam_diabetes | 380,907 | 72,386 | 28,850 | 7,975 | 2,348 | 492,466 |
| Ifam_hypertension | 302,742 | 53,537 | 21,692 | 6,096 | 1,749 | 385,816 |
| Ifam_heart_disease | 292,507 | 48,390 | 16,356 | 4,191 | 1,298 | 362,742 |
| Ifam_stroke | 187,320 | 36,508 | 14,703 | 3,784 | 1,199 | 243,514 |
| Ifam_coronary_artery_disease | 96,489 | 17,926 | 7,892 | 2,161 | 560 | 125,028 |
| Ifam_mi | 73,175 | 21,208 | 9,722 | 2,728 | 781 | 107,614 |
| Ifam_heart_attack | 82,555 | 13,446 | 4,985 | 1,060 | 287 | 102,333 |
| Ifam_arthritis | 66,376 | 11,728 | 4,739 | 1,159 | 353 | 84,355 |
| Ifam_myocardial_infarction | 62,732 | 9,387 | 3,583 | 942 | 277 | 76,921 |
| Ifam_alzheimer_s_disease | 55,178 | 9,801 | 4,090 | 1,001 | 291 | 70,361 |
| Ifam_heart_failure | 45,318 | 7,491 | 2,839 | 601 | 88 | 56,337 |
| Ifam_asthma | 43,981 | 7,949 | 3,089 | 957 | 283 | 56,259 |
| Ifam_copd | 45,035 | 7,060 | 2,820 | 832 | 281 | 56,028 |
| Ifam_htn | 30,815 | 11,111 | 4,672 | 1,213 | 306 | 48,117 |
| Ifam_dementia | 37,695 | 5,642 | 1,979 | 439 | 102 | 45,857 |
| Ifam_hyperlipidemia | 35,917 | 5,526 | 2,397 | 766 | 194 | 44,800 |
| Ifam_cad | 27,815 | 8,524 | 3,809 | 1,120 | 317 | 41,585 |
| Ifam_malignant_neoplasm | 37,904 | 1,707 | 961 | 267 | 31 | 40,870 |
| Ifam_cva | 27,780 | 7,809 | 3,409 | 1,032 | 378 | 40,408 |

# Top 20 Terms like ‘memory’

| Patient Days with Term | Index Year | | | | | |  | |
| --- | --- | --- | --- | --- | --- | --- | --- | --- |
| Term | 0 | 3 | 5 | 7 | 8 | Grand Total | |  |
| idv_memory_loss | 287,780 | 36,080 | 14,351 | 4,973 | 1,513 | 344,697 | |  |
| idv_memory_issues | 116,421 | 10,359 | 3,238 | 762 | 244 | 131,024 | |  |
| idv_memory_lapses | 49,143 | 5,114 | 1,221 | 216 | 51 | 55,745 | |  |
| idv_change_in_memory | 24,384 | 7,061 | 3,986 | 1,996 | 949 | 38,376 | |  |
| idv_memory | 28,750 | 2,646 | 1,020 | 198 | 55 | 32,669 | |  |
| idv_short_term_memory_loss | 17,211 | 862 | 265 | 57 | 18 | 18,413 | |  |
| idv_memory_disturbance | 10,709 | 207 | 55 | 26 | 8 | 11,005 | |  |
| idv_short_term_memory_issues | 7,657 | 440 | 158 | 52 | 9 | 8,316 | |  |
| idv_short_term_memory | 5,794 | 347 | 82 | 22 | 7 | 6,252 | |  |
| idv_short_term_memory_impaired | 2,995 | 136 | 27 | 4 |  | 3,162 | |  |
| Ofam_memory_issues | 2,362 | 157 | 38 | 6 |  | 2,563 | |  |
| Ofam_memory_loss | 2,017 | 109 | 42 | 14 |  | 2,182 | |  |
| idv_memory_changes | 1,891 | 164 | 58 | 14 | 2 | 2,129 | |  |
| idv_memory_for_recent_events_abnormality | 1,981 | 54 | 10 |  |  | 2,045 | |  |
| idv_memory_impaired | 1,494 | 76 | 26 | 6 |  | 1,602 | |  |
| idv_memory_dysfunction | 1,261 | 80 | 35 |  | 3 | 1,379 | |  |
| Ifam_memory_issues | 987 | 72 | 18 |  |  | 1,077 | |  |
| Ffam_memory_loss | 969 | 22 | 6 | 4 |  | 1,001 | |  |
| Ofam_memory | 873 | 57 | 18 |  |  | 948 | |  |
| Ffam_memory_issues | 879 | 37 |  | 5 |  | 921 | |  |

# Clusters

Clusters terms with at least one individual term having the subword ‘memory’ or matching ‘cognit’ are reported here. The “idv” prefix indicates this is an individual term, “Ifam” is immediate Family, “fFam” is family and “OFam” is other family.

Because these are hierarchical clusters of terms, each term is uniquely assigned to that cluster. A patient needs to only match 1 term on one day to appear in the feature for that cluster. The counts are presented for training data in the clustering phase of the analysis (all the data present in the FastText file). Patients with term are unique counts across all index years.

No postprocessing was performed to remove false associations from the clusters as this was considered too expensive to perform by hand and the project wished to evaluate the performance using only the automated results (e.g. see muscular_disease in confusion cluster).

### Confusion

| **TERM** | **Patient Days with term** | **Patients with term** |
| --- | --- | --- |
| idv_confusion | 466,757 | 188,469 |
| idv_confusional_state | 1,777 | 979 |
| idv_cognition_changes | 1,532 | 1,268 |
| idv_completed_stroke | 910 | 281 |
| idv_presenile_dementia | 666 | 170 |
| idv_cognitive_impaired | 626 | 467 |
| idv_loses_track_of_time | 500 | 420 |
| idv_mentation_issues | 485 | 409 |
| idv_muscular_disease | 483 | 323 |
| idv_cerebral_degeneration | 463 | 116 |
| idv_cerebellar_stroke_syndrome | 440 | 83 |
| idv_coordination_abnormality | 439 | 326 |
| idv_cerebral_ataxia | 382 | 70 |
| idv_mental_impairment | 367 | 286 |
| idv_viral_encephalitis | 345 | 127 |
| idv_speech_dysfunction | 293 | 211 |
| idv_aseptic_meningitis | 291 | 137 |
| idv_mumbled_speech | 278 | 239 |
| idv_grand_mal | 264 | 126 |
| idv_lack_of_attention | 259 | 235 |
| idv_delayed_speech | 257 | 210 |
| idv_motor_restlessness | 248 | 119 |
| idv_epileptic_seizure | 247 | 167 |
| idv_neurosyphilis | 233 | 125 |
| idv_meningoencephalitis | 215 | 107 |
| idv_lack_of_memory | 210 | 172 |
| idv_alcoholic_peripheral_neuropathy | 208 | 59 |
| idv_absence_seizure | 207 | 142 |
| idv_writing_changes | 206 | 183 |
| idv_ageusia | 199 | 84 |
| idv_petit_mal_seizure | 198 | 96 |
| idv_subacute_combined_degeneration | 193 | 102 |
| idv_albinism | 185 | 55 |
| idv_speech_delay | 176 | 107 |
| idv_bilateral_atrophy | 175 | 135 |
| idv_bradykinesis | 174 | 133 |
| idv_awkwardness | 173 | 136 |
| idv_echolalia | 172 | 130 |
| idv_corticobasal_degeneration | 168 | 70 |
| idv_convulsive_syncope | 168 | 104 |
| idv_cognitive_abnormality | 164 | 130 |
| idv_short_and_long_term_memory_issues | 162 | 150 |
| idv_fumbling | 162 | 145 |
| idv_empty_sella_syndrome | 160 | 58 |
| idv_facial_neuropathy | 156 | 63 |
| idv_cerebellar_disease | 155 | 118 |
| idv_papillitis | 154 | 65 |
| idv_muddled | 153 | 139 |
| idv_remembering | 153 | 138 |
| idv_frontal_lobe_syndrome | 152 | 59 |
| idv_ramsey_hunt_syndrome | 148 | 57 |
| Ffam_cognitive_impairment | 142 | 117 |
| idv_encephalomyelitis | 137 | 69 |
| Ifam_neurological_disorder | 135 | 46 |
| idv_pseudo_parkinsonism | 135 | 41 |
| Ofam_short_term_memory_issues | 134 | 125 |
| idv_petit_mal | 132 | 61 |
| idv_hemiballismus | 132 | 56 |
| idv_short_and_long_term_memory_loss | 128 | 82 |
| idv_partial_epilepsy | 127 | 43 |
| Ofam_change_in_memory | 126 | 120 |
| idv_fragile_x_syndrome | 124 | 35 |
| idv_continuous_tremor | 123 | 70 |
| Ifam_cognitive_impairment | 119 | 89 |
| idv_amnestic_syndrome | 114 | 88 |
| Ofam_cognitive_changes | 111 | 78 |
| idv_stocking_sensory_loss | 111 | 82 |
| Ofam_getting_lost | 108 | 102 |
| idv_axonal_sensorimotor_neuropathy | 107 | 53 |
| idv_driving_changes | 105 | 96 |
| idv_short_and_long_term_memory | 104 | 59 |
| Ifam_behavioral_issues | 100 | 54 |
| idv_posterior_reversible_encephalopathy_syndrome | 98 | 37 |
| idv_choreiform_movement | 97 | 49 |
| idv_upper_motor_neuron_disease | 92 | 62 |

### Memory Loss

| **TERM** | **Patient Days with term** | **Patients with term** |
| --- | --- | --- |
| idv_memory_loss | 344,697 | 140,657 |
| idv_memory_issues | 131,024 | 73,812 |
| idv_forgetful | 80,182 | 50,838 |
| idv_memory | 32,669 | 23,286 |
| idv_mild_cognitive_impairment | 24,681 | 13,337 |
| idv_short_term_memory_loss | 18,413 | 12,823 |
| idv_getting_lost | 13,490 | 11,242 |
| idv_repeat_self | 11,708 | 9,554 |
| idv_remembering_issues | 9,852 | 8,435 |
| idv_short_term_memory_issues | 8,316 | 6,802 |
| idv_forgetting_names | 8,123 | 6,838 |
| idv_misplacing_items | 7,415 | 6,253 |
| idv_short_term_memory | 6,252 | 4,485 |
| idv_mci | 4,205 | 2,912 |
| idv_recalling_issues | 3,709 | 3,194 |
| idv_lewy_body_dementia | 3,485 | 1,970 |
| idv_normal_pressure_hydrocephalus | 3,055 | 2,041 |
| idv_pseudodementia | 2,007 | 1,448 |
| idv_memory_dysfunction | 1,379 | 1,020 |
| idv_frontotemporal_dementia | 1,231 | 777 |
| idv_gait_apraxia | 722 | 372 |

### Change in Memory

| **TERM** | **Patient Days with term** | **Patients with term** |
| --- | --- | --- |
| idv_septal_deviation | 89,592 | 37,356 |
| idv_pleuritic_pain | 53,439 | 26,876 |
| idv_change_in_memory | 38,376 | 18,754 |

### Cognitive Impairment

| **TERM** | **Patient Days with term** | **Patients with term** |
| --- | --- | --- |
| idv_cognitive_impairment | 74,545 | 46,919 |
| idv_hearing_impairment | 33,270 | 18,797 |
| idv_driving_issues | 14,936 | 12,125 |

### Alzheimer’s Disease

| **TERM** | **Patient Days with term** | **Patients with term** |
| --- | --- | --- |
| idv_alzheimer_s_disease | 69,207 | 31,905 |
| Ofam_memory_issues | 2,563 | 2,261 |
| Ofam_memory_loss | 2,182 | 1,965 |
| Ofam_forgetful | 1,510 | 1,301 |
| Ifam_memory_issues | 1,077 | 940 |
| Ffam_memory_loss | 1,001 | 864 |
| Ofam_memory | 948 | 864 |
| Ffam_memory_issues | 921 | 831 |
| Ifam_memory_loss | 892 | 621 |
| Ofam_forgetting_names | 471 | 428 |

### Alertness and Cognitive Deficit

| **TERM** | **Patient Days with term** | **Patients with term** |
| --- | --- | --- |
| idv_alertness | 57,843 | 31,902 |
| idv_cognitive_deficit | 35,686 | 21,307 |
| idv_chewing_issues | 18,719 | 13,283 |
| idv_aps | 11,820 | 6,658 |
| idv_coordination | 11,419 | 7,508 |
| idv_cognition | 8,454 | 5,842 |
| idv_cognitive | 6,752 | 4,299 |

### Memory Lapses

| **TERM** | **Patient Days with term** | **Patients with term** |
| --- | --- | --- |
| idv_memory_lapses | 55,745 | 16,513 |
| idv_concentrating | 11,963 | 5,340 |

### Cognitive Issues

| **TERM** | **Patient Days with term** | **Patients with term** |
| --- | --- | --- |
| idv_cognitive_issues | 18,786 | 12,811 |
| idv_cognitive_disorder | 14,402 | 4,312 |
| idv_cognitive_decline | 13,600 | 8,190 |
| idv_cognitive_dysfunction | 8,566 | 5,506 |
| idv_cognitive_changes | 6,246 | 4,307 |

### Family History of dementia

| **TERM** | **Patient Days with term** | **Patients with term** |
| --- | --- | --- |
| Ffam_dementia | 17,038 | 6,409 |
| idv_memory_disturbance | 11,005 | 3,006 |
| Ffam_alzheimer_s_dementia | 1,243 | 432 |

### Clumsy Cognition Issues

| **TERM** | **Patient Days with term** | **Patients with term** |
| --- | --- | --- |
| idv_clumsy | 7,677 | 4,611 |
| idv_cognition_issues | 6,873 | 4,462 |
| idv_child_abuse | 4,860 | 2,680 |
| idv_elder_abuse | 4,714 | 2,800 |
| idv_gasp | 4,038 | 2,921 |
| idv_caffeine_withdrawal | 1,119 | 851 |
| idv_personality_change | 6,011 | 3,719 |
| idv_short_term_memory_impaired | 3,162 | 2,386 |
| idv_flaccidity | 2,473 | 1,501 |
| idv_muscle_rigidity | 1,584 | 820 |
| idv_long_term_memory_impaired | 891 | 605 |
| idv_hypotonicity | 774 | 473 |
| idv_naming_common_objects_issues | 660 | 420 |

### Cognition impaired

| **TERM** | **Patient Days with term** | **Patients with term** |
| --- | --- | --- |
| idv_hyperkinetic | 3,438 | 2,126 |
| idv_cognition_impaired | 1,199 | 811 |

### Memory Changes

| **TERM** | **Patient Days with term** | **Patients with term** |
| --- | --- | --- |
| idv_mentation_changes | 2,987 | 2,330 |
| idv_memory_changes | 2,129 | 1,791 |
| idv_huntington_s_disease | 1,850 | 434 |
| idv_memory_impaired | 1,602 | 1,166 |
| idv_mental_changes | 1,272 | 782 |
| idv_organizing_issues | 742 | 563 |
| idv_long_term_memory_issues | 682 | 594 |
| idv_memory_for_recent_events_impaired | 624 | 460 |
| idv_long_term_memory_loss | 563 | 450 |
| Ifam_huntington_s_disease | 489 | 162 |
| idv_learning_disorder | 465 | 320 |
| idv_lack_of_focus | 461 | 356 |
| idv_memory_for_recent_events_issues | 457 | 375 |
| idv_retrograde_amnesia | 447 | 236 |
| idv_long_term_memory | 432 | 332 |
| idv_short_term_memory_changes | 415 | 375 |
| Ofam_cognitive_impairment | 385 | 298 |
| Ffam_forgetful | 356 | 302 |
| idv_memory_for_recent_events | 335 | 303 |
| Ofam_huntington_s_disease | 323 | 104 |
| Ofam_short_term_memory_loss | 319 | 292 |
| idv_post_traumatic_amnesia | 315 | 208 |
| idv_cognitive_disturbance | 312 | 257 |
| Ffam_huntington_s_disease | 311 | 111 |
| Ofam_cognitive_decline | 299 | 225 |
| idv_forget_to_turn_off_stove | 275 | 237 |
| idv_amnestic_disorder | 263 | 141 |
| Ofam_cognitive_issues | 261 | 232 |
| idv_memory_for_remote_events_issues | 259 | 164 |
| Ifam_memory | 259 | 189 |
| idv_subjective_cognitive_impairment | 254 | 205 |
| idv_absent_minded | 248 | 227 |
| Ofam_repeat_self | 247 | 234 |
| Ffam_memory | 235 | 214 |
| Ffam_forgetting_names | 206 | 193 |
| idv_pick_s_disease | 198 | 119 |
| idv_progressive_aphasia | 159 | 98 |
| idv_echopraxia | 120 | 72 |

### Memory Abnormality

| **TERM** | **Patient Days with term** | **Patients with term** |
| --- | --- | --- |
| idv_memory_for_recent_events_abnormality | 2,045 | 1,509 |
| idv_memory_for_remote_events_abnormality | 693 | 495 |

# Odds filtering

Computing the odds ratios of features in both the matched and unmatched data is important to detect the influence of the matching. The filtering applied to the model forces variables to have a matched odds ratio less than 0.9 or greater than 1.1. More importantly codes whose ratio of unmatched odds ratio (O_U_) to matched odds ratio (O_M_) is lower than 0.5 or greater than 4 are excluded. When O_U_/O_M_ exceeds 4 this is an indication that the code has a severe skew in prevalence that the matching partially corrects. Codes that are filtered in this way include fractures of neck of femur, pathological vertebrae fractures, macular degeneration and pacemakers, among others that are most prevalent in the elderly. Codes filtered due to very low ratios are gynecological exams, lipoid screening and those that are most prevalent in the youngest population.

# Important Variables in Each model year

Tree based machine learning algorithms rank variables by gain – how much the fit improves after that feature is used in a tree node. The gain is a dimension-less value so we report the percentage of total gain attributed to each feature.

The incident model year variables are in Table 4 in the paper. Each of the following tables lists the variables that explain 80% of the total gain and where each variable explains at least 0.5% of the gain in the model for a year. In year 3, the variables that individually explain more than 0.5% of the gain explain only 52.8% of the total; in year 4 its 66.3%, in year 5 its 75.2% and in the remaining years it is more than 80%.

One reason the individual feature gains are low is the low prevalence of each of these terms in the model.

## Year 3

| Variable | Description | Percent Gain | Cumulative Gain |
| --- | --- | --- | --- |
| cls_c106 | idv_mci + idv_short_term_memory_issues | 7.3 | 7.3 |
| idv_memory_issues | memory_issues | 3.2 | 10.5 |
| cls_c186 | idv_wandering + idv_sundowning | 2.8 | 13.3 |
| idv_forgetful | forgetful | 2.2 | 15.6 |
| demog_enc | Number of Encounters | 2.2 | 17.8 |
| rxg_481 | Selective serotonin reuptake inhibitors (SSRIs) | 2.2 | 20.0 |
| idv_memory_loss | memory_loss | 2.1 | 22.1 |
| icd9_311 | Depressive Disorder, Not Elsewhere Classified | 2.0 | 24.1 |
| icd9_3320 | Paralysis Agitans | 1.9 | 26.0 |
| etg_238800 | Mood Disorder, Depressed | 1.8 | 27.8 |
| idv_dementia | dementia | 1.6 | 29.4 |
| demog_age | Age | 1.5 | 30.9 |
| idv_depression | depression | 1.4 | 32.4 |
| etg_316800 | Parkinson’s Disease | 1.3 | 33.6 |
| etg_319900 | Neurological Diseases Signs & Symptoms | 1.2 | 34.9 |
| cls_c69 | Ofam_mental_status_changes + Ofam_weak | 1.2 | 36.1 |
| rxg_124 | Atypical antipsychotics | 1.1 | 37.2 |
| cls_c21 | Ofam_tripped + Ffam_fall | 1.0 | 38.2 |
| rxg_473 | Selective serotonin / norepinephrine reuptake inhibitors (SNRIs) | 0.9 | 39.1 |
| cls_c646 | idv_cognitive_changes + idv_cognitive_decline | 0.9 | 40.0 |
| etg_239300 | Psychotic & Schizophrenic Disorders | 0.9 | 40.9 |
| ofam_informant | informant | 0.9 | 41.8 |
| cls_c10 | idv_anxiety + idv_anxiety_attack | 0.9 | 42.6 |
| cls_c141 | idv_combative + idv_delirious | 0.8 | 43.5 |
| icd9_V7612 | Other Screening Mammogram | 0.8 | 44.2 |
| cls_c304 | idv_unsteadiness + idv_unsteady_on_feet | 0.7 | 45.0 |
| idv_confused | confused | 0.7 | 45.7 |
| idv_parkinson_s_disease | parkinson_s_disease | 0.7 | 46.4 |
| icd9_78321 | Loss Of Weight | 0.7 | 47.0 |
| rxg_099 | Urinary anticholinergics | 0.6 | 47.7 |
| idv_memory | memory | 0.6 | 48.2 |
| idv_fall | fall | 0.6 | 48.8 |
| icd9_78079 | Other Malaise And Fatigue | 0.6 | 49.4 |
| idv_agitation | agitation | 0.5 | 49.9 |
| etg_238900 | Mood Disorder, Bipolar | 0.5 | 50.4 |
| icd9_56400 | Unspecified Constipation | 0.5 | 50.9 |
| idv_behavior | behavior | 0.5 | 51.4 |
| rxg_221 | Dopamine precursors, antiparkinson agents | 0.5 | 51.9 |
| idv_bipolar_disorder | bipolar_disorder | 0.5 | 52.3 |
| rxg_069 | Antidepressants, miscellaneous | 0.5 | 52.8 |

## Year 4

| Variable | Description | Percent Gain | Cumulative Gain |
| --- | --- | --- | --- |
| cls_c106 | idv_mci + idv_short_term_memory_issues | 10.5 | 10.5 |
| etg_238800 | Mood Disorder, Depressed | 4.5 | 15.0 |
| idv_memory_loss | memory_loss | 2.8 | 17.9 |
| idv_memory_issues | memory_issues | 2.6 | 20.5 |
| icd9_311 | Depressive Disorder, Not Elsewhere Classified | 2.5 | 23.0 |
| rxg_481 | Selective serotonin reuptake inhibitors (SSRIs) | 2.4 | 25.4 |
| demog_age | Age | 2.4 | 27.8 |
| demog_enc | Number of Encounters | 2.3 | 30.1 |
| idv_depression | depression | 2.1 | 32.1 |
| idv_forgetful | forgetful | 1.9 | 34.1 |
| idv_chd | chd | 1.8 | 35.9 |
| cls_c10 | idv_anxiety + idv_anxiety_attack | 1.7 | 37.6 |
| cls_c186 | idv_wandering + idv_sundowning | 1.7 | 39.3 |
| rxg_124 | Atypical antipsychotics | 1.7 | 41.0 |
| idv_parkinson_s_disease | parkinson_s_disease | 1.6 | 42.5 |
| cls_c240 | idv_rest_tremor + idv_parkinsonism | 1.4 | 44.0 |
| etg_319900 | Neurological Diseases Signs & Symptoms | 1.3 | 45.3 |
| idv_labored_breathing | labored_breathing | 1.3 | 46.6 |
| idv_dementia | dementia | 1.2 | 47.8 |
| cls_c530 | idv_cvd + idv_chd | 1.0 | 48.8 |
| cls_c197 | idv_intercostal_retractions | 1.0 | 49.8 |
| icd9_78079 | Other Malaise And Fatigue | 1.0 | 50.8 |
| icd9_78321 | Loss Of Weight | 1.0 | 51.8 |
| icd9_56400 | Unspecified Constipation | 0.9 | 52.7 |
| rxg_099 | Urinary anticholinergics | 0.9 | 53.6 |
| icd9_V7612 | Other Screening Mammogram | 0.9 | 54.5 |
| cls_c69 | Ofam_mental_status_changes + Ofam_weak | 0.9 | 55.4 |
| idv_bipolar_disorder | bipolar_disorder | 0.9 | 56.3 |
| etg_239300 | Psychotic & Schizophrenic Disorders | 0.9 | 57.1 |
| rxg_473 | Selective serotonin / norepinephrine reuptake inhibitors (SNRIs) | 0.9 | 58.0 |
| ofam_informant | informant | 0.8 | 58.8 |
| cls_c141 | idv_combative + idv_delirious | 0.8 | 59.6 |
| icd9_7804 | Dizziness And Giddiness | 0.7 | 60.3 |
| rxg_067 | Anticonvulsants, miscellaneous | 0.7 | 61.0 |
| idv_behavior | behavior | 0.6 | 61.7 |
| cls_c28 | idv_hematochezia + idv_hematuria | 0.6 | 62.3 |
| idv_agitation | agitation | 0.6 | 62.8 |
| idv_confused | confused | 0.5 | 63.4 |
| cls_c304 | idv_unsteadiness + idv_unsteady_on_feet | 0.5 | 63.9 |
| etg_316000 | Cerebral Vascular Disease | 0.5 | 64.5 |
| idv_memory | memory | 0.5 | 64.9 |
| icd9_V726 | Laboratory Examination | 0.5 | 65.4 |
| idv_shortness_of_breath | shortness_of_breath | 0.5 | 65.9 |
| etg_238900 | Mood Disorder, Bipolar | 0.5 | 66.3 |

## Year 5

| Variable | Description | Percent Gain | Cumulative Gain |
| --- | --- | --- | --- |
| cls_c106 | idv_mci + idv_short_term_memory_issues | 12.4 | 12.4 |
| etg_238800 | Mood Disorder, Depressed | 6.1 | 18.5 |
| cls_c530 | idv_cvd + idv_chd | 4.9 | 23.4 |
| idv_depression | depression | 3.7 | 27.1 |
| idv_memory_issues | memory_issues | 3.3 | 30.3 |
| idv_labored_breathing | labored_breathing | 3.2 | 33.6 |
| rxg_481 | Selective serotonin reuptake inhibitors (SSRIs) | 3.0 | 36.6 |
| idv_memory_loss | memory_loss | 2.9 | 39.5 |
| rxg_124 | Atypical antipsychotics | 2.4 | 41.9 |
| idv_chd | chd | 2.1 | 44.0 |
| demog_age | Age | 1.9 | 45.9 |
| icd9_311 | Depressive Disorder, Not Elsewhere Classified | 1.8 | 47.7 |
| cls_c240 | idv_rest_tremor + idv_parkinsonism | 1.6 | 49.3 |
| idv_forgetful | forgetful | 1.6 | 50.9 |
| cls_c10 | idv_anxiety + idv_anxiety_attack | 1.6 | 52.5 |
| cls_c186 | idv_wandering + idv_sundowning | 1.5 | 54.0 |
| etg_319900 | Neurological Diseases Signs & Symptoms | 1.3 | 55.3 |
| cls_c197 | idv_intercostal_retractions | 1.2 | 56.5 |
| demog_enc | Number of Encounters | 1.2 | 57.8 |
| idv_agitation | agitation | 1.2 | 59.0 |
| icd9_V7612 | Other Screening Mammogram | 1.1 | 60.1 |
| icd9_56400 | Unspecified Constipation | 1.1 | 61.2 |
| rxg_473 | Selective serotonin / norepinephrine reuptake inhibitors (SNRIs) | 1.0 | 62.2 |
| idv_s1 | s1 | 1.0 | 63.2 |
| rxg_099 | Urinary anticholinergics | 1.0 | 64.2 |
| idv_intercostal_retractions | intercostal_retractions | 1.0 | 65.2 |
| idv_fall | fall | 1.0 | 66.2 |
| rxg_067 | Anticonvulsants, miscellaneous | 0.9 | 67.1 |
| idv_s2 | s2 | 0.9 | 68.0 |
| cls_c141 | idv_combative + idv_delirious | 0.8 | 68.7 |
| cls_c21 | Ofam_tripped + Ffam_fall | 0.7 | 69.4 |
| idv_bipolar_disorder | bipolar_disorder | 0.7 | 70.1 |
| cls_c28 | idv_hematochezia + idv_hematuria | 0.6 | 70.7 |
| icd9_7804 | Dizziness And Giddiness | 0.6 | 71.4 |
| cls_c69 | Ofam_mental_status_changes + Ofam_weak | 0.6 | 72.0 |
| cls_c119 | idv_intermittent_tremor + Ffam_tremors | 0.6 | 72.6 |
| idv_parkinson_s_disease | parkinson_s_disease | 0.6 | 73.2 |
| cls_c147 | idv_s2 + idv_s1 | 0.5 | 73.7 |
| etg_163000 | Diabetes | 0.5 | 74.2 |
| cpt4_70450 | Computed Tomography, Head Or Brain; Without Contrast Material | 0.5 | 74.7 |
| cls_c174 | idv_hitting + idv_head_strike | 0.5 | 75.2 |

## Year 6

| Variable | Description | Percent Gain | Cumulative Gain |
| --- | --- | --- | --- |
| cls_c106 | idv_mci + idv_short_term_memory_issues | 11.0 | 11.0 |
| idv_agitation | agitation | 10.2 | 21.1 |
| idv_cvd | cvd | 9.0 | 30.1 |
| idv_depression | depression | 7.0 | 37.1 |
| cls_c530 | idv_cvd + idv_chd | 6.0 | 43.1 |
| etg_238800 | Mood Disorder, Depressed | 5.8 | 48.9 |
| idv_metabolic_syndrome | metabolic_syndrome | 3.4 | 52.3 |
| idv_s2 | s2 | 3.3 | 55.6 |
| idv_memory_loss | memory_loss | 2.5 | 58.1 |
| icd9_311 | Depressive Disorder, Not Elsewhere Classified | 2.4 | 60.4 |
| cls_c512 | idv_insulin_resistance_syndrome + idv_insulin_resistance | 2.2 | 62.6 |
| cls_c10 | idv_anxiety + idv_anxiety_attack | 2.1 | 64.8 |
| cls_c197 | idv_intercostal_retractions | 2.0 | 66.8 |
| idv_s1 | s1 | 2.0 | 68.8 |
| cls_c21 | Ofam_tripped + Ffam_fall | 1.6 | 70.4 |
| rxg_481 | Selective serotonin reuptake inhibitors (SSRIs) | 1.6 | 72.0 |
| cls_c71 | idv_lives_with_relations_next_door + idv_lives_with_relations_nearby | 1.3 | 73.3 |
| demog_age | Age | 1.3 | 74.6 |
| idv_intercostal_retractions | intercostal_retractions | 1.1 | 75.7 |
| idv_behavior | behavior | 1.0 | 76.7 |
| idv_fall | fall | 1.0 | 77.7 |
| idv_anxiety | anxiety | 0.9 | 78.7 |
| cls_c16 | idv_breath_sounds + idv_respiratory_distress | 0.8 | 79.4 |
| rxg_067 | Anticonvulsants, miscellaneous | 0.8 | 80.2 |

## Year 7

| Variable | Description | Percent Gain | Cumulative Gain |
| --- | --- | --- | --- |
| idv_s1 | s1 | 43.3 | 43.3 |
| cls_c91 | idv_accessory_muscle_use + idv_fremitus | 18.7 | 62.0 |
| cls_c106 | idv_mci + idv_short_term_memory_issues | 13.4 | 75.3 |
| idv_anxiety | anxiety | 8.5 | 83.9 |

## Year 8

| Variable | Description | Percent Gain | Cumulative Gain |
| --- | --- | --- | --- |
| cls_c197 | idv_intercostal_retractions | 39.8 | 39.8 |
| idv_varicosities | varicosities | 25.5 | 65.2 |
| idv_joint_swelling | joint_swelling | 8.5 | 73.8 |
| icd9_25000 | Diabetes Mellitus Without Mention Of Complication, Type Ii Or Unspecified Type, Not Stated As Uncontrolled | 6.5 | 80.2 |
